# Prenatal Risk Factors and Perinatal and Postnatal Outcomes Associated With Maternal Opioid Exposure in an Urban, Low-Income, Multiethnic US Population

**DOI:** 10.1001/jamanetworkopen.2019.6405

**Published:** 2019-06-28

**Authors:** Romuladus E. Azuine, Yuelong Ji, Hsing-Yuan Chang, Yoona Kim, Hongkai Ji, Jessica DiBari, Xiumei Hong, Guoying Wang, Gopal K. Singh, Colleen Pearson, Barry Zuckerman, Pamela J. Surkan, Xiaobin Wang

**Affiliations:** 1 Division of Research, Office of Epidemiology and Research, Maternal and Child Health Bureau, Health Resources and Services Administration, US Department of Health and Human Services, Rockville, Maryland; 2 Center on Early Life Origins of Disease, Department of Population, Family, and Reproductive Health, Johns Hopkins University Bloomberg School of Public Health, Baltimore, Maryland; 3 Department of International Health, Johns Hopkins University Bloomberg School of Public Health, Baltimore, Maryland; 4 Department of Biostatistics, Johns Hopkins University Bloomberg School of Public Health, Baltimore, Maryland; 5 Office of Health Equity, Health Resources and Services Administration, US Department of Health and Human Services , Rockville, Maryland; 6 Department of Pediatrics, Boston University School of Medicine, Boston Medical Center, Boston, Massachusetts; 7 Division of General Pediatrics and Adolescent Medicine, Department of Pediatrics, Johns Hopkins University School of Medicine, Baltimore, Maryland

## Abstract

**Importance:**

The opioid epidemic increasingly affects pregnant women and developing fetuses, resulting in high rates of neonatal abstinence syndrome. However, longitudinal studies that prospectively observe newborns with neonatal abstinence syndrome or with maternal opioid use and examine their long-term physical and neurodevelopmental outcomes are lacking.

**Objective:**

To examine prenatal risk factors associated with maternal opioid use during pregnancy and the short-term and long-term health consequences on their children.

**Design, Setting, and Participants:**

This cohort study analyzed data from the Boston Birth Cohort, an urban, low-income, multiethnic cohort that enrolled mother-newborn pairs at birth at Boston Medical Center (Boston, Massachusetts) starting in 1998, and a subset of children were prospectively observed at Boston Medical Center pediatric primary care and subspecialty clinics from birth to age 21 years. Data analysis began in June 2018 and was completed in May 2019.

**Exposures:**

In utero opioid exposure was defined as maternal self-reported opioid use and/or clinical diagnosis of neonatal abstinence syndrome.

**Main Outcomes and Measures:**

Pregnancy outcomes, postnatal child physical health, and major neurodevelopmental disabilities, documented in maternal and child medical records.

**Results:**

This study included 8509 Boston Birth Cohort mother-newborn pairs for prenatal and perinatal analyses. Of those, 3153 children continued to receive pediatric care at Boston Medical Center and were included in assessing postnatal outcomes. Overall, 454 of the 8509 children (5.3%) in the Boston Birth Cohort had in utero opioid exposure. At birth, opioid exposure was associated with higher risks of fetal growth restriction (odds ratio [OR], 1.87; 95% CI, 1.41-2.47) and preterm birth (OR, 1.49; 95% CI, 1.19-1.86). Opioid exposure was associated with increased risks of lack of expected physiological development (OR, 1.80; 95% CI, 1.17-2.79) and conduct disorder/emotional disturbance (OR, 2.13; 95% CI, 1.20-3.77) among preschool-aged children. In school-aged children, opioid exposure was associated with a higher risk of attention-deficit/hyperactivity disorder (OR, 2.55; 95% CI, 1.42-4.57).

**Conclusions and Relevance:**

In this sample of urban, high-risk, low-income mother-child pairs, in utero opioid exposure was significantly associated with adverse short-term and long-term outcomes across developmental stages, including higher rates of physical and neurodevelopmental disorders in affected children. Efforts to prevent the opioid epidemic and mitigate its health consequences would benefit from more intergenerational research.

## Key Points

**Question:**

What are the prenatal risk factors and perinatal and postnatal outcomes associated with maternal opioid use during pregnancy?

**Findings:**

In this cohort study based on data from 8509 mother-child pairs in the Boston Birth Cohort, in utero opioid exposure was significantly associated with higher risks of fetal growth restriction, preterm birth, lack of expected physiological development, childhood conduct disorder or emotional disturbance in preschool-aged children, and attention-deficit/hyperactivity disorder in school-aged children.

**Meaning:**

Prenatal opioid exposure was associated with higher risks of adverse perinatal and postnatal physical health and neurodevelopmental outcomes, suggesting that efforts to mitigate the health consequences of the opioid epidemic require more intergenerational research.

## Teaser

This cohort study examines the prenatal risk factors and perinatal and postnatal outcomes associated with maternal opioid use during pregnancy.

## Introduction

In the last 25 years, the United States has experienced a dramatic increase in the already epidemic use of opioids, resulting in unprecedented levels of overdose, opioid use disorder, and other harms related to opioids.^1,2^ Nationwide, the rate of opioid use disorder among women delivering newborns more than quadrupled between 1999 and 2014.^3^ Opioid drug use during pregnancy, whether heroin or prescription painkillers, harms the health of mother and child.^4,5,6,7^ A major consequence is opioid-associated neonatal abstinence syndrome (NAS), defined by signs of withdrawal that infants develop after in utero exposure to opioids.^8^ Newborns with severe NAS require inpatient pharmacologic intervention, with a mean length of hospitalization of 16 days in the United States.^9,10,11^

Maternal opioid use can have a negative effect on exposed mothers and their children, which may fuel an opioid epidemic that spans multiple generations. Leading governmental and professional associations, including the US Department of Health and Human Services, the American College of Obstetricians and Gynecologists, and the American Academy of Pediatrics, are making concerted efforts to identify risk factors and improve prevention strategies to reduce the negative health effects of opioids on pregnant women and mitigate the developmental consequences of in utero opioid exposure.^2,12,13^ Neonatal abstinence syndrome is also part of the United States’ national strategy to combat the opioid epidemic, encapsulated in the National Institutes of Health Helping to End Addiction Over the Long-term initiative.^14^

However, many questions related to in utero opioid exposure remain unanswered. Significant knowledge gaps exist regarding the long-term consequences of maternal opioid use on physical health and neurodevelopmental outcomes among children exposed to opioids. Foremost, there is a lack of large, prospective birth cohort studies to assess the long-term impact of in utero opioid exposure, in part owing to the need for a long duration of follow-up, the risk of attrition, complicated logistics, and the large expense associated with sustaining longitudinal studies. There is a recognized disparity in opioid-related data available on urban low-income minority populations and historically understudied populations in the United States.

Prior studies^15,16,17^ have reported an increased risk of chronic health conditions, prolonged hospitalizations, unintended pregnancies, and psychopathology among women exposed or addicted to opioids. Whiteman et al^17^ have also reported an increased risk of threatened preterm labor and early-onset delivery among these women. Meanwhile, among infants and children exposed to opioids in utero, studies^17,18,19,20,21,22,23,24^ report preterm birth and stillbirth, hypoxia, low mental and psychomotor development, language and reading deficits, low cognitive development and functioning, lower IQ score, neurodevelopmental impairment, and early marijuana use. Tolia et al^25^ found that NAS was associated with the increasing amount of resources related to neonatal intensive care units from 2004 to 2013.^25^ There is a trend in the incidence of NAS and its related health care expenditures between 2000 and 2009 in the United States^26^ and between 2000 and 2011 in Australia.^27^ Haight et al^3^ reported a 333% increase in opioid use disorder in terms of delivery hospitalizations in the United States.

While each study offers important contributions, most are dated, used small sample sizes, were conducted in different social-cultural settings, or relied on maternal self-reports for identification of child perinatal and postnatal outcomes. To our knowledge, no longitudinal cohort studies of urban, low-income US residents to date have examined maternal sociodemographic and epidemiological characteristics associated with opioid use or perinatal and postnatal outcomes of children exposed to opioids. Understanding the characteristics of pregnant women and the factors that potentially put them at risk of opioid exposure or opioid use disorder and opioids’ long-term effect on their children is important for developing and implementing policies and programs to address the opioid epidemic burden among pregnant women and prevent adverse outcomes in their children.

In this study, we conducted a longitudinal analysis of the risks and consequences of maternal opioid exposure, using a rich longitudinal database of mother-newborn pairs included in the Boston Birth Cohort (BBC), a large sample of an urban, low-income, multiethnic population in the United States. Specifically, we examined (1) demographic and epidemiological characteristics of mothers with opioid exposure compared with mothers without exposure, (2) prenatal risk factors of maternal opioid exposure, (3) pregnancy complications and birth outcomes of mother-newborn pairs with opioid exposure compared with their counterparts without exposure, and (4) the postnatal physical health and neurodevelopmental outcomes of children with opioid exposure compared with children without exposure.

## Methods

### Study Design and Setting

A detailed description of the study cohort has been previously published.^28,29^ Briefly, eligible mothers were those who delivered singleton live births at Boston Medical Center (BMC), Boston, Massachusetts, starting in 1998. The enrollment targeted singleton, live-preterm-birth (<37 weeks’ gestation), low-birth-weight infants (<2500 g), and full-term, normal-weight infants, with oversampling of preterm and/or low-birth-weight babies. Multiple-gestation pregnancies (eg, twins, triplets), newborns with major birth defects, birth by in vitro fertilization, and infants with congenital chromosomal abnormalities were excluded from the cohort. Among those enrolled at birth, 3153 mother-newborn pairs who continued to receive pediatric primary care at BMC were enrolled in a postnatal follow-up study.^28,30,31^ The institutional review boards of Boston University Medical Center and the Johns Hopkins Bloomberg School of Public Health approved the study protocol. This report follows the Strengthening the Reporting of Observational Studies in Epidemiology (STROBE) reporting guideline.

### Data Collection Procedures

After obtaining written informed consent from the enrolled participants, trained research staff conducted face-to-face interviews 48 to 72 hours after delivery using a standardized questionnaire to determine sociodemographic variables, illicit drug use patterns, and smoking and drinking status. Pregnancy complications, birth outcomes, and diagnoses for NAS were obtained via maternal and infant medical record abstraction. Starting in 2003, postnatal growth and developmental outcomes were collected from electronic medical records (EMRs) as part of routine clinical data collection. For each primary care visit, EMRs contain codes for primary and secondary physician diagnoses corresponding to the *International Statistical Classification of Diseases, Ninth Revision, Clinical Modification (ICD-9-CM) *(before October 1, 2015) and *Tenth Revision *(*ICD*-*10*) (after October 1, 2015).

### Definition of Maternal Characteristics

Pertinent maternal sociodemographic information was collected using standard maternal questionnaires, including maternal age, self-reported height and prepregnancy body weight, number of live births, annual household income, race/ethnicity, marital status, and education status. Body mass index (BMI) before pregnancy was calculated as prepregnancy body weight in kilograms divided by height in meters squared. In addition, maternal questionnaires collected information on cigarette smoking status, alcohol use, and illicit drug use. Illicit drugs were categorized into 3 groups: (1) marijuana, (2) stimulants, and (3) opioids. Stimulants included cocaine, crack, ecstasy, amphetamines, and phencyclidine. Opioids included heroin, oxycodone, and methadone. The duration of ever smoking, drinking, and drug use was defined as the period from 6 months before pregnancy to delivery.

### Definition of NAS

Neonatal abstinence syndrome was defined for infants with a hospital diagnosis of *ICD*-*9* code 779.5 (drug withdrawal syndrome in newborn) or *ICD*-*10* code P96.1 (neonatal withdrawal symptoms from maternal use of drugs of addiction). Diagnoses were documented in medical records.

### Definition of Perinatal and Postnatal Outcomes

This study’s major outcomes were pregnancy complications, birth outcomes, and postnatal physical health and neurodevelopmental disabilities. The following pregnancy complications were defined based on maternal medical records: prepregnancy BMI categorized as underweight (<18.5), normal weight (18.5-25), or overweight (≥25) according to the Institute of Medicine BMI classification^32^; maternal diabetes was defined as having either gestational or pregestational diabetes^33^; and hypertensive disorder was classified yes if preeclampsia, eclampsia, chronic hypertension, and/or hemolysis, elevated liver enzymes, and low platelets syndrome were present during pregnancy.^34^ Birth outcomes, including birth weight, gestational age, and fetal growth pattern, were obtained from maternal and child medical records. Gestational age was assessed based on the first day of the last menstrual period and early prenatal ultrasonographic results, as described previously.^29^ Preterm birth was defined as gestational age less than 37 weeks. Low birth weight was defined as less than 2500 g.^29^ Fetal growth was defined by birth weight for gestational age and categorized into 3 groups: (1) small for gestational age (<10th percentile), (2), appropriate for gestational age (10th-90th percentile), and (3) large for gestational age (>90th percentile), according to an established local sex- and race-specific reference population living in the Boston area.^35^

Postnatal physical health and neurodevelopmental disabilities were defined according to *ICD*-*9* and *ICD*-*10* codes from study children’s EMRs. They included being ever diagnosed with attention-deficit/hyperactivity disorder (ADHD) (*ICD*-*9*: 314.0-314.9; *ICD*-*10*: F90.0-F90.9), conduct disorder/emotional disturbance (*ICD*-*9*: 312.0-312.9, 313.0-313.9; *ICD*-*10*: F91.0-F91.9, F93.0, F93.8, F93.9), and lack of expected normal physiological development (*ICD*-9: 783.40-783.43; *ICD*-*10*: R62.0, R62.50-R62.59).

### Statistical Analysis

The incidence rates for NAS from 2003 to 2016 were calculated and plotted as a bar chart using each year’s enrolled newborns and NAS diagnoses from the EMRs (eFigure in the Supplement). Missing data for sociodemographic characteristics were imputed using multiple imputation by chained equations with the predictive mean matching method. Women were classified into 2 groups defined by NAS diagnosis and maternal self-reported opioids use as follows: (1) the exposed to opioids group was defined as having an NAS diagnosis or maternal self-reported opioids use and (2) the nonexposed group was defined as not having an NAS diagnosis and no maternal self-reported opioids use. Univariate analyses were used to compare baseline characteristics of mothers for maternal opioid exposure, using full data, complete-case data (only participants without any missing data), and multiple imputed data. For categorical data, χ^2^ tests (or Fisher exact tests for small samples) were used; for continuous data, *t* tests were used. Baseline characteristics of mother-infant pairs in the BBC follow-up were compared with those of mother-infant pairs not in the follow-up using univariate analysis. Pregnancy complications, birth, and postnatal physical health and neurodevelopmental outcomes between the 2 groups were compared using crude and multiple logistic regression analyses. The following sociodemographic variables were adjusted for pregnancy complications and birth outcomes in multiple logistic regression: maternal age, household income, race/ethnicity, marital status, and maternal education. For children’s postnatal physical health and neurodevelopmental outcomes, preterm birth and low-birth-weight conditions were also adjusted in the multiple logistic regression. The postnatal physical health and neurodevelopmental outcomes were further examined by child age groups (<6 years vs ≥6 years). Categorical outcomes were analyzed using multinomial logistic regression. The following sensitivity analyses were conducted in this study: (1) log-linear regression for pregnancy complications, birth, and postnatal physical health and neurodevelopmental outcomes, (2) Cox regression model for risk of ADHD diagnosis, (3) logistic regression for pregnancy complications, birth, and postnatal physical health and neurodevelopmental outcomes after excluding infants born before 2003, and (4) logistic regression for pregnancy complications and birth outcomes further adjusting birth year period (1998-2007 vs 2008-2014 and later). The pooled estimates of 10 imputed data sets were used for all analytical results using multiple imputed data according to Rubin rule.^36^ All analyses were performed using R version 3.4.3 (The R Foundation). Statistical significance was set at *P* < .05, and all tests were 2-tailed.

## Results

### Incidence of NAS

Of the 8509 mother-newborn pairs enrolled in the BBC, 454 infants (5.3%) were prenatally exposed to opioids (Figure). There was an upward trend in NAS incidence over the last 15 years of data, ranging from a low of 12.1 per 1000 hospital births in 2003 to a high of 63.1 per 1000 hospital births in 2012 (eFigure in the Supplement). The incidence of NAS in the cohort remained more than 24 per 1000 hospital births starting in 2004, but it increased to 60.9 per 1000 in 2008 and continued to be higher than 32 per 1000 through 2016. The incidence rates were not calculated from 1998 to 2002 owing to the unavailability of EMRs.

**Figure.**
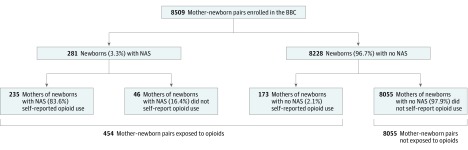
Flowchart of 8509 Mother-Newborn Pairs Enrolled in the Boston Birth Cohort (BBC) NAS indicates neonatal abstinence syndrome.

### Baseline Maternal Characteristics

Table 1 shows the baseline epidemiological and drug use characteristics of mothers in the opioid exposed and nonexposed groups in the BBC, using full data, complete-case data, and multiple imputed data. In our data, 1201 of 8509 records (14.9%) were incomplete, mainly owing to missing maternal BMI (579 participants [6.8%]). All other study variables were more than 96% complete. As shown in Table 1, there were no substantial differences in the results between the complete-case analyses and multiple imputation. The mothers exposed to opioids were more likely to be older, have lower BMI, have more than 1 child, be non-Hispanic white, be unmarried, have less than a college education, and have higher rates of smoking, drinking, and using other illicit drugs during pregnancy compared with their nonexposed counterparts. Among 454 mothers with opioid exposure, 348 (76.7%) reported heroin use, and 204 (44.9%) reported oxycodone use. Additionally, 46 mothers (10.1%) whose exposure to opioids was evidenced by NAS diagnosis did not report any opioid use. The baseline characteristics of mother-newborn pairs were compared between children in the BBC follow-up vs nonobserved children (eTable 1 in the Supplement). The 2 groups were generally comparable except for minor differences in sociodemographic variables and lower percentage of stimulant and opioid use.

**Table 1.** Baseline Characteristics of Mothers in the Boston Birth Cohort

Baseline Characteristic	No. (%)								
	Full Data			Complete-Case Data^a^			Multiple Imputed Data^b^		
	Nonexposed	Exposed to Opioids^c^	*P* Value^d^	Nonexposed	Exposed to Opioids^c^	*P* Value^d^	Nonexposed	Exposed to Opioids^c^	*P* Value^d^
No.	8055	454	NA	6854	427	NA	8055	454	NA
Maternal age, y									
≤20	900 (11.2)	9 (2.0)	<.001	781 (11.4)	9 (2.1)	<.001	900 (11.2)	9 (2.0)	<.001
21-30	4036 (50.1)	267 (58.8)		3402 (49.6)	253 (59.3)		4036 (50.1)	267 (58.8)	
>30	3119 (38.7)	178 (39.2)		2671 (39.0)	165 (38.6)		3119 (38.7)	178 (39.2)	
Maternal prepregnancy BMI, mean (SD)	26.1 (6.3)	24.4 (5.6)	<.001	26.1 (6.3)	24.3 (5.4)	<.001	26.1 (6.3)	24.3 (5.6)	<.001
Missing	571 (7.1)	8 (1.8)	NA	0	0	NA	0	0	NA
Live births									
1	3484 (43.3)	166 (36.6)	.005	3019 (44.0)	161 (37.7)	.01	3495 (43.4)	167 (36.8)	.007
>1	4553 (56.5)	285 (62.8)		3835 (56.0)	266 (62.3)		4560 (56.6)	287 (63.2)	
Missing	18 (0.2)	3 (0.7)	NA	0	0	NA	0	0	NA
Household income, $									
<30 000	3287 (40.8)	200 (44.1)	.39	2826 (41.2)	190 (44.5)	.33	3287 (40.8)	200 (44.1)	.39
≥30 000	1039 (12.9)	55 (12.1)		944 (13.8)	51 (11.9)		1039 (12.9)	55 (12.1)	
Unknown	3729 (46.3)	199 (43.8)	NA	3084 (45.0)	186 (43.6)	NA	3729 (46.3)	199 (43.8)	NA
Race/Ethnicity									
Black	4339 (53.9)	54 (11.9)	<.001	3719 (54.3)	51 (11.9)	<.001	4339 (53.9)	54 (11.9)	<.001
Non-Hispanic white	684 (8.5)	322 (70.9)		588 (8.6)	304 (71.2)		684 (8.5)	322 (70.9)	
Hispanic	2367 (29.4)	56 (12.3)		1979 (28.9)	51 (11.9)		2367 (29.4)	56 (12.3)	
Other^e^	665 (8.3)	22 (4.8)		568 (8.3)	21 (4.9)		665 (8.3)	22 (4.8)	
Marital status									
Married	2801 (34.8)	70 (15.4)	<.001	2416 (35.2)	66 (15.5)	<.001	2861 (35.5)	72 (15.9)	<.001
Not married	5090 (63.2)	379 (83.5)		4438 (64.8)	361 (84.5)		5194 (64.5)	382 (84.1)	
Missing	164 (2.0)	5 (1.1)	NA	0	0	NA	0	0	NA
Maternal education									
<College	5108 (63.4)	315 (69.4)	.03	4334 (63.2)	301 (70.5)	.003	5206 (64.6)	319 (70.3)	.02
≥College	2792 (34.7)	134 (29.5)		2520 (36.8)	126 (29.5)		2849 (35.4)	135 (29.7)	
Missing	155 (1.9)	5 (1.1)	NA	0	0	NA	0	0	NA
Maternal smoking									
No	6731 (83.6)	74 (16.3)	<.001	5720 (83.5)	66 (15.5)	<.001	6789 (84.3)	74 (16.3)	<.001
Yes	1256 (15.6)	380 (83.7)		1134 (16.5)	361 (84.5)		1266 (15.7)	380 (83.7)	
Missing	68 (0.8)	0	NA	0	0	NA	0	0	NA
Maternal drinking									
No	7092 (88.0)	373 (82.2)	<.001	6252 (91.2)	355 (83.1)	<.001	7372 (91.5)	377 (83.0)	<.001
Yes	660 (8.2)	76 (16.7)		602 (8.8)	72 (16.9)		683 (8.5)	77 (17.0)	
Missing	303 (3.8)	5 (1.1)	NA	0	0	NA	0	0	NA
Child’s sex									
Female	4035 (50.1)	237 (52.2)	.41	3417 (49.9)	223 (52.2)	.37	4035 (50.1)	237 (52.2)	.41
Male	4020 (49.9)	217 (47.8)		3437 (50.1)	204 (47.8)		4020 (49.9)	217 (47.8)	
Maternal marijuana use									
No	6746 (83.7)	113 (24.9)	<.001	5639 (82.3)	100 (23.4)	<.001	6746 (83.7)	113 (24.9)	<.001
Yes	1309 (16.3)	341 (75.1)		1215 (17.7)	327 (76.6)		1309 (16.3)	341 (75.1)	
Maternal stimulants use									
Any stimulant use									
No	7852 (97.5)	133 (29.3)	<.001	6669 (97.3)	122 (28.6)	<.001	7852 (97.5)	133 (29.3)	<.001
Yes	203 (2.5)	321 (70.7)		185 (2.7)	305 (71.4)		203 (2.5)	321 (70.7)	
Cocaine use									
No	7899 (98.1)	153 (33.7)	<.001	6712 (97.9)	142 (33.3)	<.001	7899 (98.1)	153 (33.7)	<.001
Yes	156 (1.9)	301 (66.3)		142 (2.1)	285 (66.7)		156 (1.9)	301 (66.3)	
Crack use									
No	8010 (99.4)	264 (58.1)	<.001	6815 (99.4)	246 (57.6)	<.001	8010 (99.4)	264 (58.1)	<.001
Yes	45 (0.6)	190 (41.9)		39 (0.6)	181 (42.4)		45 (0.6)	190 (41.9)	
Amphetamine use									
No	8032 (99.7)	388 (85.5)	<.001	6834 (99.7)	364 (85.2)	<.001	8032 (99.7)	388 (85.5)	<.001
Yes	23 (0.3)	66 (14.5)		20 (0.3)	63 (14.8)		23 (0.3)	66 (14.5)	
Ecstasy use									
No	8012 (99.5)	308 (67.8)	<.001	6814 (99.4)	288 (67.4)	<.001	8012 (99.5)	308 (67.8)	<.001
Yes	43 (0.5)	146 (32.2)		40 (0.6)	139 (32.6)		43 (0.5)	146 (32.2)	
PCP use									
No	8050 (99.9)	427 (94.1)	<.001	6849 (99.9)	401 (93.9)	<.001	8050 (99.9)	427 (94.1)	<.001
Yes	5 (0.1)	27 (5.9)		5 (0.1)	26 (6.1)		5 (0.1)	27 (5.9)	
Maternal opioid use									
Any opioid use									
No	8055 (100.0)	46 (10.1)	<.001	6854 (100.0)	43 (10.1)	<.001	8055 (100)	46 (10.1)	<.001
Yes	0	408 (89.9)		0	384 (89.9)		0	408 (89.9)	
Heroin use									
No	8055 (100.0)	106 (23.3)	<.001	6854 (100.0)	99 (23.2)	<.001	8055 (100)	106 (23.3)	<.001
Yes	0	348 (76.7)		0	328 (76.8)		0	348 (76.7)	
Methadone use									
No	8055 (100.0)	210 (46.3)	<.001	6854 (100.0)	194 (45.4)	<.001	8055 (100)	210 (46.3)	<.001
Yes	0	244 (53.7)		0	233 (54.6)		0	244 (53.7)	
Oxycodone use									
No	8055 (100.0)	250 (55.1)	<.001	6854 (100.0)	233 (54.6)	<.001	8055 (100)	250 (55.1)	<.001
Yes	0	204 (44.9)		0 (0.0)	194 (45.4)		0 (0.0)	204 (44.9)	

Abbreviations: BMI, body mass index (calculated as weight in kilograms divided by height in meters squared); NA, not applicable; PCP, phencyclidine.

^a^ Complete case refers to only using data from patients without any missing data.

^b^ Pooled estimates of 10 imputed data sets.

^c^ Opioid exposure is defined as mothers with self-reported opioid use during pregnancy or with a newborn diagnosed with neonatal abstinence syndrome based on electronic medical records.

^d^ The *P* values were obtained by χ^2^ test (or Fisher exact test for small samples) for categorical variables and *t* tests for continuous variables.

^e^ Other category included Asian, Pacific Islander, Caribbean, multiracial, and others.

### Pregnancy Complications and Birth Outcomes

Pregnancy complications and birth outcomes between the opioid-exposed group and the nonexposed group are presented in Table 2. For pregnancy complications, the opioid-exposed group had a significantly higher probability of having diabetes (odds ratio [OR], 1.64; 95% CI, 1.19-2.26) and, with normal weight as a reference, having a lower BMI level (OR, 0.60; 95% CI, 0.47-0.75) compared with the nonexposed group. For birth outcomes, there was a significantly higher probability of preterm birth (OR, 1.49; 95% CI, 1.19-1.86) and being small for gestational age (OR, 1.87; 95% CI, 1.41-2.47) for infants exposed to opioids. Sensitivity analysis results using log-linear regression (eTable 2 in the Supplement), excluding infants born before 2003 (eTable 5 in the Supplement), and logistic regression further adjusting birth year period (eTable 7 in the Supplement) yielded comparable point estimates and confidence intervals.

**Table 2.** Pregnancy Complications and Birth Outcomes Associated With Maternal Opioid Exposure in the Boston Birth Cohort

Outcome	Multiple Imputation^a^	
	Crude Logistic Regression, OR (95% CI)	Adjusted Logistic Regression, OR (95% CI)^b^
**Pregnancy Complications**		
Hypertensive disorder		
No	1 [Reference]	1 [Reference]
Yes	0.72 (0.52-0.99)	0.92 (0.65-1.31)
Maternal diabetes		
No	1 [Reference]	1 [Reference]
Yes	1.42 (1.08-1.87)	1.64 (1.19-2.26)
Prepregnancy weight status^c^		
Normal weight	1 [Reference]	1 [Reference]
Underweight	1.36 (0.94-1.97)	1.22 (0.78-1.89)
Overweight	0.52 (0.42-0.64)	0.60 (0.47-0.75)
**Birth Outcome**		
Preterm birth		
No	1 [Reference]	1 [Reference]
Yes	1.60 (1.31-1.95)	1.49 (1.19-1.86)
Fetal growth		
AGA	1 [Reference]	1 [Reference]
LGA	0.47 (0.29-0.75)	0.41 (0.25-0.68)
SGA	1.84 (1.46-2.33)	1.87 (1.41-2.47)

Abbreviations: AGA, appropriate for gestational age; LGA, large for gestational age; OR, odds ratio; SGA, small for gestational age.

^a^ Pooled estimates of 10 imputed data sets included 8509 mother-child pairs.

^b^ Multiple logistic regression adjusted for maternal age, household income, race/ethnicity, marital status, and maternal education.

^c^ Categorized as underweight (body mass index [calculated as weight in kilograms divided by height in meters squared] <18.5), normal weight (body mass index 18.5-25), or overweight (body mass index ≥25) according to the Institute of Medicine body mass index classification.^32^

### Postnatal Physical Health and Neurodevelopmental Outcomes

Table 3 presents postnatal physical health and neurodevelopmental outcomes in overall sample and by age group (<6 years vs ≥6 years). For children with medical records for their first 6 years, children exposed to opioids had a higher percentage of diagnoses of conduct disorder/emotional disturbance (OR, 2.13; 95% CI, 1.20-3.77) and lack of expected normal physiological development (OR, 1.80; 95% CI, 1.17-2.79) compared with children without exposure. Among children with follow-up records after age 6 years, children exposed to opioids had a higher percentage of diagnoses of ADHD (OR, 2.55; 95% CI, 1.42-4.57) compared with their counterparts. The sensitivity analysis results using log-linear regression (eTable 3 in the Supplement), Cox regression model for risk of ADHD diagnosis (eTable 4 in the Supplement), and logistic regression excluding infants born before 2003 (eTable 6 in the Supplement) yielded comparable point estimates and confidence intervals.

**Table 3.** Postnatal Physical Health and Neurodevelopmental Outcomes Associated With Maternal Opioid Exposure in the Boston Birth Cohort^a^

Outcome	Multiple Imputation OR (95% CI)^b^	
	Crude Logistic Regression	Adjusted Logistic Regression^c^
**All Age Groups, 3153 Mother-Child Pairs**		
ADHD		
No	1 [Reference]	1 [Reference]
Yes	1.28 (0.82-2.01)	1.30 (0.78-2.18)
Conduct disorder or emotional disturbance		
No	1 [Reference]	1 [Reference]
Yes	1.37 (0.9-2.07)	1.48 (0.91-2.4)
Lack of expected normal physiological development		
No	1 [Reference]	1 [Reference]
Yes	2.18 (1.54-3.1)	2.06 (1.34-3.17)
**Age <6 y, 3106 Mother-Child Pairs**		
ADHD		
No	1 [Reference]	1 [Reference]
Yes	1.60 (0.82-3.11)	1.01 (0.46-2.23)
Conduct disorder or emotional disturbance		
No	1 [Reference]	1 [Reference]
Yes	2.17 (1.35-3.49)	2.13 (1.20-3.77)
Lack of expected normal physiological development		
No	1 [Reference]	1 [Reference]
Yes	1.88 (1.33-2.66)	1.80 (1.17-2.79)
**Age ≥6 y, 2391 Mother-Child Pairs**		
ADHD		
No	1 [Reference]	1 [Reference]
Yes	2.86 (1.67-4.91)	2.55 (1.42-4.57)
Conduct disorder or emotional disturbance		
No	1 [Reference]	1 [Reference]
Yes	2.00 (1.11-3.59)	1.79 (0.95-3.35)
Lack of expected normal physiological development		
No	1 [Reference]	1 [Reference]
Yes	0.96 (0.43-2.12)	0.62 (0.26-1.46)

Abbreviations: ADHD, attention-deficit/hyperactivity disorder; OR, odds ratio.

^a^ Based on *International Classification of Diseases, Ninth Revision, Clinical Modification *(*ICD-9*) and *ICD*-*10* diagnosis codes: ADHD (*ICD*-*9*: 314.0-314.9; *ICD*-*10*: F90.0-F90.9), conduct disorder or emotional disturbance (*ICD*-*9*: 312.0-312.9, 313.0-313.9; *ICD*-*10*: F91.0-F91.9, F93.0, F93.8, F93.9), and lack of expected normal physiological development (*ICD*-*9*: 783.40-783.43; *ICD*-*10*: R62.0, R62.50-R62.59).

^b^ Pooled estimates of 10 imputed data sets.

^c^ Multiple logistic regression adjusted for maternal age, household income, race/ethnicty, marital status, maternal education, preterm birth, and low birth weight.

## Discussion

To our knowledge, this is the first study to describe the risk factors and perinatal and postnatal outcomes of maternal opioid exposure. To do this, we used a rich longitudinal database of mother-newborn pairs included in the BBC, a large sample of an urban, low-income, multiethnic population in the United States. We found that NAS incidence in the BBC increased drastically from 12.1 per 1000 hospital births to more than 30 per 1000 hospital births over the past 15 years. This rising trend is consistent with the national trend, although the BBC rate was much higher compared with national data from 2009 to 2012.^9^ A US Centers for Disease Control and Prevention *Morbidity and Mortality Weekly Report* article shows that NAS incidence by US state in 2013 ranged from 0.7 cases per 1000 hospital births (Hawaii) to 33.4 cases per 1000 hospital births (West Virginia).^10^ Because our sample was a predominantly urban, low-income, minority cohort, the higher incidence rates in our study underscore the potential disparities in NAS experienced by urban low-income populations living in the inner cities of the United States. Our higher rates may partly be attributed to the implementation of Project Recovery, Empowerment, Social Services, Prenatal Care, Education, Community, and Treatment (RESPECT) at BMC in 2006.^37^ This program identified and treated pregnant women with substance use disorder and their children, who were subsequently delivered at BMC.

In addition to a higher rate of opioid use, our study demonstrates that mothers exposed to opioids had a higher likelihood of polysubstance use, including alcohol consumption, cigarette smoking, and stimulant use. They were also more likely to be multiparous, be non-Hispanic white, be unmarried, and have less than a college education. These findings regarding the risk factors of opioid use and consequences of NAS are consistent with previous literature.^17^ They also lend further credence to the previous finding that opioid use is associated with multiple deleterious exposures, such as low socioeconomic status, poor nutrition, and other substance use disorders, which were highlighted in an executive summary of a joint workshop by the major federal and national public health agencies and assocaitons.^13^

Furthermore, we found that infants’ exposure to opioids was associated with lower gestational age, lower birth weight, and higher rate of being small for gestational age, consistent with previous literature on NAS and birth outcomes.^13,38^ More importantly, our study fills a major gap in the knowledge base regarding the long-term postnatal outcomes associated with maternal opioid exposure. Our examination of the association of maternal exposure to opioids with major physical health and neurodevelopmental outcomes for children younger and older than 6 years is noteworthy. We found that within the first 6 years, opioid exposure was associated with a higher risk of diagnosis of conduct disorder/emotional disturbance and lack of expected normal physiological development. After 6 years, opioid exposure was associated with higher likelihood of a diagnosis of ADHD. Taken together, our data suggests that prenatal opioid exposure was associated with higher risk of different yet interrelated adverse perinatal and postnatal physical health and neurodevelopmental outcomes unfolding over the life course. This differential risk over the life course may explain the inconsistent postnatal outcome findings from a previous prenatal drug exposure study.^39^

If confirmed, the findings of this study could have multiple clinical and public health implications. First, most mothers with opioid exposure have polysubstance use in addition to opioids. Thus, opioid addiction is only part of the problem. The root causes and other substance use should be addressed simultaneously. Second, children born from mothers exposed to opioids had a higher risk of physical and mental health problems along the life course, including poor fetal growth, low birth weight, preterm birth, conduct disorder/emotional disturbance, lack of expected normal physiological development, and ADHD. Additionally, maternal exposure to opioids together with poor birth outcomes may further increase the risk of adverse childhood physical health and neurodevelopmental outcomes. Thus, efforts to prevent maternal opioid use and mitigate its health consequences require a longitudinal approach that addresses physical and mental health sequelae for mothers and children. Finally, from a health economics perspective, the opioid epidemic is an intergenerational problem. Successfully addressing it will not only benefit current and future generations but also mitigate exorbitant health care expenditures.

### Strengths and Limitations

This study has several strengths. First, the prospective birth cohort design of the study made it possible to investigate early life factors before the onset of opioid-associated NAS and the long-term postnatal outcomes of maternal opioid exposure. This longitudinal approach helped us to better understand the temporal associations of opioids exposure, NAS, and their consequences later in life. Additionally, to our knowledge, BBC is among the largest contemporary prospective US birth cohorts, which allowed us to investigate multiple potential postnatal outcomes. Second, this study used physician diagnoses extracted from EMRs to identify a wide range of postnatal physical and neurodevelopmental outcomes, which is rarely found in the literature.^20,21,22,23^ This type of data is more reliable than maternal self-reported outcomes, which were used by many prior studies. Third, the study sample is mainly from a low-income, urban, minority population in the Boston area. This is a high-risk yet understudied population. We showed that this sample had a NAS rate that was almost 10-fold that of the general population, which further underscores the well-noted need for expanded research and programmatic interventions within these populations.

This study has some limitations. First, it may be subject to exposure misclassification. We defined exposure heterogeneously to include illicit, therapeutic, and unknown opioid exposures leading to NAS. This heterogeneity in exposure could lead to differential effects on long-term child outcomes, which are difficult to disentangle in this study. Second, our analyses of long-term associations might be confounded by other pregnancy exposures listed in Table 1 and other unmeasured postnatal confounders. Third, the degree to which participants lost to follow-up may have resulted in an underestimation of the diagnoses of postnatal outcomes for children exposed to opioids is unknown. Further, this study mainly consisted of a low-income, urban, minority sample, which limits the generalizability of our findings. Thus, our findings warrant additional studies.

## Conclusions

In this sample of an urban, low-income, multiethnic US population, the prevalence of NAS was almost 10-fold the national rate. Most mothers who were exposed to opioids used other substances, including marijuana, stimulants, cigarettes, and alcohol. Maternal opioid exposure was associated with a higher risk of pregnancy complications and poor birth outcomes as well as adverse postnatal child physical health and neurodevelopmental outcomes. However, the effect of opioids is still difficult to disentangle from effects of other childhood exposures. Policy and programmatic efforts to prevent NAS and mitigate its health consequences require more comprehensive longitudinal and intergenerational research.

## Notes

Supplement. **eFigure.** Trend of the Incidence Rate for Physician Diagnosed Neonatal Abstinence Syndrome (NAS) in the Boston Birth Cohort, Based on Electronic Medical Record Data From 2003 to 2016 **eTable 1.** Baseline Characteristics of Mother-Newborn Pairs With Postnatal Follow-up vs Those Without in the Boston Birth Cohort, Using 1 of 10 Imputed Data Sets **eTable 2.** Pregnancy Complications and Birth Outcomes Associated With Maternal Opioid Exposure in the Boston Birth Cohort, With Complete Data Results, Using Log-Linear Model **eTable 3.** Postnatal Physical Health and Neurodevelopmental Outcomes Associated With Maternal Opioid Exposure in the Boston Birth Cohort, With Complete Data Results, Using Log-Linear Model **eTable 4.** ADHD Associated With Maternal Opioid Exposure in the Boston Birth Cohort, Using Cox Regression Model **eTable 5.** Pregnancy Complications and Birth Outcomes Associated With Maternal Opioid Exposure After Excluding Infants Born Before 2003 **eTable 6.** Postnatal Physical Health and Neurodevelopmental Outcomes Associated With Maternal Opioid Exposure After Excluding Infants Born Before 2003 **eTable 7.** Pregnancy Complications and Birth Outcomes Associated With Maternal Opioid Exposure, Further Adjusting Birth Year Period (1998-2007 vs 2008-2014+)
