# Supplementary material for: Prenatal Risk Factors and Perinatal and Postnatal Outcomes Associated With Maternal Opioid Exposure in an Urban, Low-Income, Multiethnic US Population
Source: JAMA Netw Open. 2019 Jun 28;2(6):e196405. doi: 10.1001/jamanetworkopen.2019.6405 (PMC6604084; doi:10.1001/jamanetworkopen.2019.6405)
Supplement: Supplement. — eFigure. Trend of the Incidence Rate for Physician Diagnosed Neonatal Abstinence Syndrome (NAS) in the Boston Birth Cohort, Based on Electronic Medical Record Data From 2003 to 2016 eTable 1. Baseline Characteristics of Mother-Newborn Pairs With Postnatal Follow-up vs Those Without in the Boston Birth Cohort, Using 1 of 10 Imputed Data Sets eTable 2. Pregnancy Complications and Birth Outcomes Associated With Maternal Opioid Exposure in the Boston Birth Cohort, With Complete Data Results, Using Log-Linear Model eTable 3. Postnatal Physical Health and Neurodevelopmental Outcomes Associated With Maternal Opioid Exposure in the Boston Birth Cohort, With Complete Data Results, Using Log-Linear Model eTable 4. ADHD Associated With Maternal Opioid Exposure in the Boston Birth Cohort, Using Cox Regression Model eTable 5. Pregnancy Complications and Birth Outcomes Associated With Maternal Opioid Exposure After Excluding Infants Born Before 2003 eTable 6. Postnatal Physical Health and Neurodevelopmental Outcomes Associated With Maternal Opioid Exposure After Excluding Infants Born Before 2003 eTable 7. Pregnancy Complications and Birth Outcomes Associated With Maternal Opioid Exposure, Further Adjusting Birth Year Period (1998-2007 vs 2008-2014+) [file jamanetwopen-2-e196405-s001.pdf]

Supplementary Online Content

Azuine RE, Ji Y, Chang H-Y, et al. Prenatal risk factors and perinatal and postnatal outcomes associated with maternal opioid exposure in an urban, low-income, multiethnic US population. *JAMA Netw Open*. 2019;2(6):e196405. doi:10.1001/jamanetworkopen.2019.6405

eFigure. Trend of the Incidence Rate for Physician Diagnosed Neonatal Abstinence Syndrome (NAS) in the Boston Birth Cohort, Based on Electronic Medical Record Data From 2003 to 2016

eTable 1. Baseline Characteristics of Mother-Newborn Pairs With Postnatal Follow-up vs Those Without in the Boston Birth Cohort, Using 1 of 10 Imputed Data Sets

eTable 2. Pregnancy Complications and Birth Outcomes Associated With Maternal Opioid Exposure in the Boston Birth Cohort, With Complete Data Results, Using Log-Linear Model

eTable 3. Postnatal Physical Health and Neurodevelopmental Outcomes Associated With Maternal Opioid Exposure in the Boston Birth Cohort, With Complete Data Results, Using Log-Linear Model

eTable 4. ADHD Associated With Maternal Opioid Exposure in the Boston Birth Cohort, Using Cox Regression Model

eTable 5. Pregnancy Complications and Birth Outcomes Associated With Maternal Opioid Exposure After Excluding Infants Born Before 2003

eTable 6. Postnatal Physical Health and Neurodevelopmental Outcomes Associated With Maternal Opioid Exposure After Excluding Infants Born Before 2003

eTable 7. Pregnancy Complications and Birth Outcomes Associated With Maternal Opioid Exposure, Further Adjusting Birth Year Period (1998-2007 vs 2008-2014+)

This supplementary material has been provided by the authors to give readers additional information about their work.

© 2019 Azuine RE et al. *JAMA Network Open.*

eFigure. Trend of the Incidence Rate for Physician Diagnosed Neonatal Abstinence Syndrome (NAS) in the Boston Birth Cohort, Based on Electronic Medical Record Data From 2003 to 2016

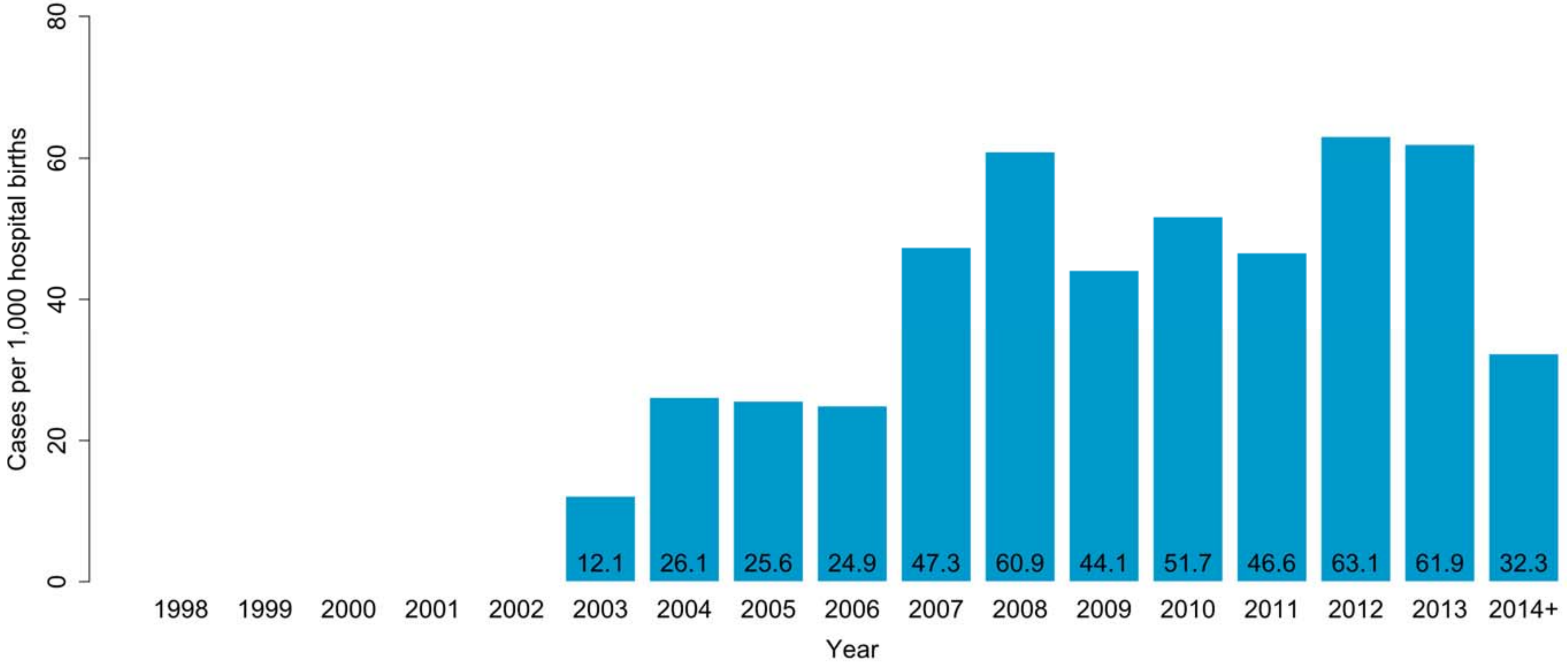

Year	1998	1999	2000	2001	2002	2003	2004	2005	2006	2007	2008	2009	2010	2011	2012	2013	2014+
Total birth	36	543	455	598	445	497	498	391	522	676	706	703	716	687	666	339	31
NAS	NA	NA	NA	NA	NA	6	13	10	13	32	43	31	37	32	42	21	1

Note: EMR data was not available before 2003.

© 2019 Azuine RE et al. *JAMA Network Open.*

eTable 1. Baseline Characteristics of Mother-Newborn Pairs With Postnatal Follow-up vs. Those Without in the Boston Birth Cohort, Using 1 of 10 Imputed Data Sets

Baseline characteristics		With Postnatal f/u	No Postnatal f/u	P-value
N		3153	5356	
Maternal age (%)				
	<=20	295 (9.4)	614 (11.5)	0.003
	21-30	1584 (50.2)	2719 (50.8)	
	>30	1274 (40.4)	2023 (37.8)	
Maternal pre-pregnancy BMI (mean (SD))		27 (6.6)	26 (6.0)	<0.001
Parity (%)				
	Nulliparous	1344 (42.6)	2318 (43.3)	0.572
	Multiparous	1809 (57.4)	3038 (56.7)	
Household income (%)				
	<$30,000	1353 (42.9)	2134 (39.8)	0.020
	>=$30,000	388 (12.3)	706 (13.2)	
	Unknown	1412 (44.8)	2516 (47)	
Ethnicity (%)				
	Black	2011 (63.8)	2382 (44.5)	<0.001
	White	228 (7.2)	778 (14.5)	
	Hispanic	702 (22.3)	1721 (32.1)	
	Others	212 (6.7)	475 (8.9)	
Marital status (%)				
	Married	1041 (33)	1892 (35.3)	0.032
	Not Married	2112 (67)	3464 (64.7)	
Maternal education (%)				
	Below college	2026 (64.3)	3499 (65.3)	0.328
	Above college	1127 (35.7)	1857 (34.7)	
Maternal smoking (%)				
	No	2572 (81.6)	4291 (80.1)	0.106
	Yes	581 (18.4)	1065 (19.9)	
Maternal drinking (%)				
	No	2896 (91.8)	4853 (90.6)	0.058
	Yes	257 (8.2)	503 (9.4)	
Child's gender (%)				
	Female	1564 (49.6)	2708 (50.6)	0.407
	Male	1589 (50.4)	2648 (49.4)	
**Maternal marijuana use (%)**				
	No	2538 (80.5)	4321 (80.7)	0.860
	Yes	615 (19.5)	1035 (19.3)	
**Maternal stimulants use (%)**				
	No	3007 (95.4)	4978 (92.9)	<0.001
	Yes	146 (4.6)	378 (7.1)	
Cocaine (%)				
	No	3024 (95.9)	5028 (93.9)	<0.001
	Yes	129 (4.1)	328 (6.1)	
Crack (%)				
	No	3078 (97.6)	5196 (97)	0.113
	Yes	75 (2.4)	160 (3)	
Amphetamine (%)				
	No	3121 (99)	5299 (98.9)	0.916
	Yes	32 (1)	57 (1.1)	
Ecstasy (%)				
	No	3095 (98.2)	5225 (97.6)	0.079

© 2019 Azuine RE et al. *JAMA Network Open.*

	Yes	58 (1.8)	131 (2.4)	
Phencyclidine (PCP) (%)				
	No	3144 (99.7)	5333 (99.6)	0.387
	Yes	9 (0.3)	23 (0.4)	
**Maternal Opioids use (%)**				
	No	3035 (96.3)	5066 (94.6)	0.001
	Yes	118 (3.7)	290 (5.4)	
Heroin (%)				
	No	3054 (96.9)	5107 (95.4)	0.001
	Yes	99 (3.1)	249 (4.6)	
Methadone (%)				
	No	3082 (97.7)	5183 (96.8)	0.011
	Yes	71 (2.3)	173 (3.2)	
Oxycodone (%)				
	No	3080 (97.7)	5225 (97.6)	0.759
	Yes	73 (2.3)	131 (2.4)	
NAS baby (%)				
	No	3063 (97.1)	5165 (96.4)	0.087
	Yes	90 (2.9)	191 (3.6)	

Note: The p-values were obtained by chi-squared (or fisher exact for small cells) tests and t-tests for categorical and continuous variables, respectively. The frequency, percentage, and p-value are pooled estimations from ten imputed dataset.

© 2019 Azuine RE et al. *JAMA Network Open.*

eTable 2. Pregnancy Complications and Birth Outcomes Associated With Maternal Opioid Exposure in the Boston Birth Cohort, With Complete Data Results, Using Log-Linear Model

Outcomes		Complete-case analysis (N=7281) §				Multiple imputation (N=8509) ‡			
		Crude Log-linear		Adjusted Log-linear †		Crude Log-linear		Adjusted Log-linear †	
		RR	95% CI	RR	95% CI	RR	95% CI	RR	95% CI
N									
**Pregnancy complications**									
Hypertensive disorder (%)									
	No	Reference		Reference		Reference		Reference	
	Yes	0.74	[0.54,1.00]	0.95	[0.67,1.32]	0.74	[0.55,1.01]	0.93	[0.67,1.30]
Maternal diabetes (%)									
	No	Reference		Reference		Reference		Reference	
	Yes	1.36	[1.03,1.75]	1.53	[1.12,2.06]	1.36	[1.05,1.76]	1.54	[1.15,2.06]
Prepregnancy weight status (%)*									
	Underweight	1.22	[0.83,1.74]	1.13	[0.71,1.76]	1.32	[0.93,1.86]	1.16	[0.76,1.76]
	Normal weight	Reference		Reference		Reference		Reference	
	Overweight/obese	0.68	[0.57,0.80]	0.73	[0.60,0.88]	0.69	[0.58,0.81]	0.75	[0.62,0.90]
**Birth outcomes**									
Preterm birth (%)									
	No	Reference		Reference		Reference		Reference	
	Yes	1.32	[1.11,1.55]	1.26	[1.03,1.52]	1.38	[1.18,1.61]	1.30	[1.09,1.57]
Fetal growth (%)¶									
	AGA	Reference		Reference		Reference		Reference	
	LGA	0.50	[0.30,0.78]	0.45	[0.26,0.72]	0.50	[0.31,0.78]	0.44	[0.27,0.72]
	SGA	1.72	[1.39,2.12]	1.72	[1.32,2.22]	1.65	[1.34,2.04]	1.66	[1.29,2.13]

*According to the Institute of Medicine's BMI classification.
§Complete-case means only use the data without any missing.
¶ AGA: appropriate for gestational age; LGA: large for gestational age; SGA: small for gestational age
†Multiple Log-linear Regression adjusted for: maternal age, household income, race, marital status, maternal education
‡pooled estimates of ten imputed datasets

© 2019 Azuine RE et al. *JAMA Network Open.*

eTable 3. Postnatal Physical Health and Neurodevelopmental Outcomes Associated with Maternal Opioid Exposure in the Boston Birth Cohort, With Complete Data Results, Using Log-Linear Model

Outcomes		Complete-case analysis (N=7281)*				Multiple imputation (N=8509)‡			
		Crude Log-linear		Adjusted Log-linear †		Crude Log-linear		Adjusted Log-linear †	
		RR	95% CI	RR	95% CI	RR	95% CI	RR	95% CI
**<u>All age groups</u>**		N=2798				N=3153			
ADHD									
	No	Reference		Reference		Reference		Reference	
	Yes	1.28	[0.83,1.87]	1.30	[0.79,2.05]	1.23	[0.82,1.84]	1.24	[0.78,1.98]
Conduct disorder or Emotional disturbance									
	No	Reference		Reference		Reference		Reference	
	Yes	1.35	[0.91,1.91]	1.44	[0.92,2.00]	1.28	[0.89,1.85]	1.37	[0.89,2.10]
Lack of expected normal physiological development									
	No	Reference		Reference		Reference		Reference	
	Yes	1.52	[1.19,1.91]	1.39	[1.04,1.82]	1.52	[1.20,1.92]	1.38	[1.05,1.80]
**<u>Medical record within first 5 years</u>**		N=2767				N=3106			
ADHD									
	No	Reference		Reference		Reference		Reference	
	Yes	1.64	[0.80,2.96]	1.09	[0.48,2.31]	1.55	[0.82,2.95]	1.00	[0.47,2.16]
Conduct disorder or Emotional disturbance									
	No	Reference		Reference		Reference		Reference	
	Yes	2.00	[1.26,3.03]	1.99	[1.14,3.31]	1.97	[1.28,3.05]	1.93	[1.14,3.26]
Lack of expected normal physiological development									
	No	Reference		Reference		Reference		Reference	
	Yes	1.45	[1.11,1.85]	1.35	[0.99,1.80]	1.45	[1.13,1.86]	1.33	[1.00,1.78]
**<u>Medical record at 6 and older</u>**		N=2107				N=2391			
ADHD									
	No	Reference		Reference		Reference		Reference	
	Yes	2.21	[1.40,3.32]	2.01	[1.21,3.17]	2.17	[1.41,3.33]	1.97	[1.23,3.15]
Conduct disorder or Emotional disturbance									
	No	Reference		Reference		Reference		Reference	
	Yes	1.82	[1.06,2.91]	1.61	[0.89,2.72]	1.73	[1.05,2.85]	1.57	[0.91,2.70]
Lack of expected normal physiological development									
	No	Reference		Reference		Reference		Reference	
	Yes	0.96	[0.41,1.87]	0.67	[0.28,1.37]	0.96	[0.45,2.03]	0.67	[0.31,1.48]

© 2019 Azuine RE et al. *JAMA Network Open.*

§Based on ICD-9 and ICD-10 diagnosis codes: ADHD (ICD-9: 314.0-314.9; ICD-10: F90.0-F90.9), conduct disorder or emotional disturbance (ICD-9: 312.0-312.9, 313.0-313.9; ICD-10: F91.0-F91.9, F93.0, F93.8, F93.9), and lack of expected normal physiological development (ICD-9: 783.40-783.43; ICD-10: R62.0, R62.50-R62.59).
*Complete-case means only use the data without any missing.
†Multiple Log-linear Regression adjusted for: maternal age, household income, race, marital status, maternal education, preterm birth, low birthweight.
‡pooled estimates of ten imputed datasets

© 2019 Azuine RE et al. *JAMA Network Open.*

eTable 4. ADHD Associated With Maternal Opioid Exposure in the Boston Birth Cohort, Using Cox Regression Model

Outcomes		Complete-case analysis (N=7281)*				Multiple imputation (N=8509)‡			
		Crude Cox model		Adjusted Cox model†		Crude Cox model		Adjusted Cox model†	
		HR	95% CI	HR	95% CI	HR	95% CI	HR	95% CI
All age groups			N=2798				N=3153		
ADHD	No	Reference		Reference		Reference		Reference	
	Yes	2.50	[1.66,3.74]	2.11	[1.33,3.35]	2.46	[1.64,3.68]	2.07	[1.32,3.24]
Follow-up length < 6			N=691				N=762		
ADHD	No	Reference		Reference		Reference		Reference	
	Yes	2.45	[0.53,11.41]	0.69	[0.10,4.81]	1.99	[0.44,8.91]	0.81	[0.13,5.19]
Follow-up length ≥6 years			N=2107				N=2391		
ADHD	No	Reference		Reference		Reference		Reference	
	Yes	2.60	[1.71,3.96]	2.28	[1.42,3.65]	2.59	[1.7,3.94]	2.23	[1.41,3.55]

†Cox regression adjusted for: maternal age, household income, race, marital status, maternal education, preterm birth, low birthweight.
*Complete-case means only use the data without any missing.
‡pooled estimates of ten imputed datasets

© 2019 Azuine RE et al. *JAMA Network Open.*

eTable 5. Pregnancy Complications and Birth Outcomes Associated With Maternal Opioid Exposure After Excluding Infants Born Before 2003

Outcomes		Complete-case analysis (N=5700)§				Multiple imputation (N=6432)‡			
		Crude logistic		Adjusted logistic†		Crude logistic		Adjusted logistic†	
		OR	95% CI	OR	95% CI	OR	95% CI	OR	95% CI
N									
**Pregnancy complications**									
Hypertensive disorder (%)									
	No	Reference		Reference		Reference		Reference	
	Yes	0.73	[0.51,1.01]	0.99	[0.66,1.46]	0.74	[0.53,1.03]	0.98	[0.67,1.44]
Maternal diabetes (%)									
	No	Reference		Reference		Reference		Reference	
	Yes	1.46	[1.08,1.94]	1.69	[1.18,2.40]	1.34	[1.03,1.75]	1.52	[1.11,2.09]
Prepregnancy weight status (%)*									
	Underweight	1.45	[0.97,2.17]	1.32	[0.79,2.20]	1.56	[1.06,2.30]	1.31	[0.80,2.15]
	Normal weight	Reference		Reference		Reference		Reference	
	Overweight/obese	0.50	[0.40,0.63]	0.59	[0.45,0.77]	0.49	[0.39,0.61]	0.58	[0.44,0.75]
**Birth outcomes**									
Preterm birth (%)									
	No	Reference		Reference		Reference		Reference	
	Yes	1.50	[1.20,1.86]	1.42	[1.09,1.84]	1.35	[1.14,1.61]	1.30	[1.06,1.60]
Fetal growth (%)¶									
	AGA	Reference		Reference		Reference		Reference	
	LGA	0.46	[0.27,0.78]	0.39	[0.22,0.69]	0.43	[0.25,0.73]	0.38	[0.21,0.66]
	SGA	1.93	[1.49,2.51]	1.85	[1.33,2.57]	1.85	[1.43,2.39]	1.76	[1.27,2.42]

*According to the Institute of Medicine's BMI classification.
§Complete-case means only use the data without any missing.
¶ AGA: appropriate for gestational age; LGA: large for gestational age; SGA: small for gestational age
†Multiple logistic regression adjusted for: maternal age, household income, race, marital status, maternal education.
‡pooled estimates of ten imputed datasets

eTable 6. Postnatal Physical Health and Neurodevelopmental Outcomes§ Associated With Maternal Opioid Exposure After Excluding Infants Born Before 2003

Outcomes		Complete-case analysis (N=5700)*				Multiple imputation (N=6432)‡			
		Crude logistic		Adjusted logistic†		Crude logistic		Adjusted logistic†	
		OR	95% CI	OR	95% CI	OR	95% CI	OR	95% CI
**All age groups**		N=2380				N=2613			
ADHD									
	No	Reference		Reference		Reference		Reference	
	Yes	1.31	[0.79,2.09]	1.33	[0.73,2.32]	1.27	[0.78,2.06]	1.27	[0.72,2.24]
Conduct disorder or Emotional disturbance									
	No	Reference		Reference		Reference		Reference	
	Yes	1.32	[0.82,2.04]	1.37	[0.79,2.32]	1.26	[0.80,1.98]	1.26	[0.74,2.13]
Lack of expected normal physiological development									
	No	Reference		Reference		Reference		Reference	
	Yes	2.08	[1.44,3.02]	2.15	[1.34,3.45]	2.02	[1.40,2.90]	1.97	[1.24,3.13]
**Medical record within first 5 years**		N=2378				N=2610			
ADHD									
	No	Reference		Reference		Reference		Reference	
	Yes	1.19	[0.49,2.43]	0.80	[0.29,1.94]	1.14	[0.52,2.49]	0.76	[0.30,1.91]
Conduct disorder or Emotional disturbance									
	No	Reference		Reference		Reference		Reference	
	Yes	1.79	[1.04,2.91]	1.75	[0.92,3.21]	1.75	[1.05,2.90]	1.67	[0.90,3.07]
Lack of expected normal physiological development									
	No	Reference		Reference		Reference		Reference	
	Yes	1.81	[1.26,2.61]	1.81	[1.13,2.91]	1.76	[1.23,2.53]	1.70	[1.07,2.70]
**Medical record at 6 and older**		N=1703				N=1866			
ADHD									
	No	Reference		Reference		Reference		Reference	
	Yes	3.19	[1.75,5.67]	2.77	[1.44,5.21]	3.15	[1.76,5.63]	2.66	[1.41,5.02]
Conduct disorder or Emotional disturbance									
	No	Reference		Reference		Reference		Reference	
	Yes	2.15	[1.09,3.99]	1.81	[0.86,3.59]	2.07	[1.09,3.95]	1.71	[0.85,3.44]
Lack of expected normal physiological development									
	No	Reference		Reference		Reference		Reference	
	Yes	0.72	[0.25,1.67]	0.52	[0.17,1.29]	0.71	[0.28,1.81]	0.49	[0.18,1.32]

§Based on ICD-9 and ICD-10 diagnosis codes: ADHD (ICD-9: 314.0-314.9; ICD-10: F90.0-F90.9), conduct disorder or emotional disturbance (ICD-9: 312.0-312.9, 313.0-313.9; ICD-10: F91.0-F91.9, F93.0, F93.8, F93.9), and lack of expected normal physiological development (ICD-9: 783.40-783.43; ICD-10: R62.0, R62.50-R62.59).
*Complete-case means only use the data without any missing.
†Multiple logistic regression adjusted for: maternal age, household income, race, marital status, maternal education, preterm birth, low birthweight.
‡pooled estimates of ten imputed datasets

© 2019 Azuine RE et al. *JAMA Network Open.*

eTable 7. Pregnancy Complications and Birth Outcomes Associated With Maternal Opioid Exposure, Further Adjusting Birth Year Period (1998-2007 Vs 2008-2014+)

Outcomes		Complete-case analysis (N=7281)§				Multiple imputation (N=8509)‡			
		Crude logistic		Adjusted logistic†		Crude logistic		Adjusted logistic†	
		OR	95% CI	OR	95% CI	OR	95% CI	OR	95% CI
N									
**Pregnancy complications**									
Hypertensive disorder (%)									
	No	Reference		Reference		Reference		Reference	
	Yes	0.71	[0.51,0.98]	0.93	[0.64,1.33]	0.72	[0.52,0.99]	0.91	[0.64,1.30]
Maternal diabetes (%)									
	No	Reference		Reference		Reference		Reference	
	Yes	1.42	[1.06,1.87]	1.58	[1.12,2.19]	1.42	[1.08,1.87]	1.57	[1.14,2.17]
Prepregnancy weight status (%)*									
	Underweight	1.25	[0.84,1.84]	1.16	[0.72,1.85]	1.36	[0.94,1.97]	1.18	[0.76,1.85]
	Normal weight	Reference		Reference		Reference		Reference	
	Overweight/obese	0.51	[0.42,0.64]	0.57	[0.45,0.73]	0.52	[0.42,0.64]	0.58	[0.46,0.73]
**Birth outcomes**									
Preterm birth (%)									
	No	Reference		Reference		Reference		Reference	
	Yes	1.50	[1.22,1.84]	1.45	[1.14,1.84]	1.60	[1.31,1.95]	1.51	[1.21,1.90]
Fetal growth (%)¶									
	AGA	Reference		Reference		Reference		Reference	
	LGA	0.48	[0.29,0.77]	0.43	[0.25,0.71]	0.47	[0.29,0.75]	0.42	[0.25,0.68]
	SGA	1.94	[1.53,2.47]	1.97	[1.47,2.64]	1.84	[1.46,2.33]	1.88	[1.42,2.50]

*According to the Institute of Medicine's BMI classification.
§Complete-case means only use the data without any missing.
¶ AGA: appropriate for gestational age; LGA: large for gestational age; SGA: small for gestational age
†Multiple logistic regression adjusted for: maternal age, household income, race, marital status, maternal education, birth year period (1998-2007 vs 2008-2014+).
‡pooled estimates of ten imputed datasets

© 2019 Azuine RE et al. *JAMA Network Open.*
